# ASD v3.0: unraveling allosteric regulation with structural mechanisms and biological networks

**DOI:** 10.1093/nar/gkv902

**Published:** 2015-09-13

**Authors:** Qiancheng Shen, Guanqiao Wang, Shuai Li, Xinyi Liu, Shaoyong Lu, Zhongjie Chen, Kun Song, Junhao Yan, Lv Geng, Zhimin Huang, Wenkang Huang, Guoqiang Chen, Jian Zhang

**Affiliations:** 1 Department of Pathophysiology, Key Laboratory of Cell Differentiation and Apoptosis of Chinese Ministry of Education, Shanghai Jiao-Tong University School of Medicine (SJTU-SM), Shanghai 200025, China; 2 General Surgery Department, Renji hospital Shanghai Jiao-Tong University School of Medicine (SJTU-SM), Shanghai 200127, China; 3 Medicinal Bioinformatics Center, Shanghai Jiao-Tong University School of Medicine (SJTU-SM), Shanghai 200025, China

## Abstract

Allosteric regulation, the most direct and efficient way of regulating protein function, is induced by the binding of a ligand at one site that is topographically distinct from an orthosteric site. Allosteric Database (ASD, available online at http://mdl.shsmu.edu.cn/ASD) has been developed to provide comprehensive information featuring allosteric regulation. With increasing data, fundamental questions pertaining to allostery are currently receiving more attention from the mechanism of allosteric changes in an individual protein to the entire effect of the changes in the interconnected network in the cell. Thus, the following novel features were added to this updated version: (i) structural mechanisms of more than 1600 allosteric actions were elucidated by a comparison of site structures before and after the binding of an modulator; (ii) 261 allosteric networks were identified to unveil how the allosteric action in a single protein would propagate to affect downstream proteins; (iii) two of the largest human allosteromes, protein kinases and GPCRs, were thoroughly constructed; and (iv) web interface and data organization were completely redesigned for efficient access. In addition, allosteric data have largely expanded in this update. These updates are useful for facilitating the investigation of allosteric mechanisms, dynamic networks and drug discoveries.

## INTRODUCTION

Allosteric regulation fine-tunes most biological processes, including signal transduction, enzyme activity, metabolism and transport (1–3). Allostery, an intrinsic property of a protein, is referred to as the regulation of activity at one site (also known as an orthosteric site) in a protein by a topographically and spatially distant site; the latter is designated as an allosteric site (4–6). Allosteric regulation occurs through binding of a modulator (e.g., small molecule) at an allosteric site to engender a conformational change that affects function at the orthosteric site (7–11). This effect may cause the re-distribution of the conformational ensemble by either stabilizing an active conformation or destabilizing an inactive conformation in response to allosteric perturbations (12,13). Traditionally, the repertoire of allostery was primarily confined to determining the allosteric effects or mechanisms in individual multi-subunit or monomer proteins by conformational transitions (14–17). Recently, increasing evidence has indicated that allosteric signals can propagate across several or numerous proteins to sculpt allosteric networks (18–22). A quintessential example of allosteric propagation is pertinent to the identification of interconnected proteins that govern the reversible switch between gluconeogenesis and glycolysis in human metabolite dynamics (18). Thus, focusing on the fundamental role of allostery in a cellular network is an instrumental strategy for dissecting the consequences of pathological allosteric events (7,20–22).

Despite the increasing interest in the development of allosteric drugs as a new tactic in drug discovery, the structural mechanisms underlying allosteric drug action represent a key challenge. The hotspots of conformational changes in the allosteric site are of particular importance to understanding the underpinnings underlying allostery, because the efficacy of an allosteric drug is often determined by specific conformational transitions in the allosteric site (23). Identification of triggering motions in an allosteric site is highly helpful to uncovering structural mechanisms of allosteric drug action and contributes to drug discovery.

In drug discovery, protein kinases and G protein-coupled receptors (GPCRs) are two of the largest targets. Due to the remarkable structural conservation of orthosteric sites in the kinome and homologous GPCRs, there has been a long-standing challenge in the development of highly specific kinase and GPCR orthosteric inhibitors or activators (24–27). Fortunately, breakthroughs in GPCR structural determination have resulted in a fast-growing number of GPCR structures obtained in complex with allosteric modulators (28–34), and biochemical studies, such as disulfide trapping (35), high-throughput screening (36) and fragment-based screening (37), promote the identification of allosteric sites in protein kinases. Thus, elucidating the family profile of allosteric sites in protein kinases and GPCRs provides a promising new paradigm for the discovery of novel allosteric sites and the design of potent modulators with improved protein subtype selectivity, adverse effects and pathway-biased signaling (38–43).

The Allosteric Database (ASD) has been developed to provide comprehensive information characterizing allosteric regulation since 2009 (44,45), ranging from allosteric proteins, modulators to interactions, sites, pathways, functions and related diseases. Here, in a current update of the resource, in addition to the significant expansion of data, we focused on the characterization of structural mechanisms of allosteric drug action, allosteric networks, and protein kinases and GPCRs allosteromes. These updates include the elucidation of >1600 allosteric actions in 308 allosteric proteins, the identification of 261 endogenous/exogenous allosteric networks and the construction of protein kinases and GPCRs allosterome. In addition, the web interface and data organization have been completely redesigned for efficient access on the basis of community demands. Cumulatively, these updates have the benefit of prompting an investigation of allosteric mechanism, allosteric related disease and allosteric drug discovery.

## DATABASE GROWTH AND STATISTICS

Allosteric molecules and features, including structures, sites, pathways, functions, related diseases and external links, were collected using previously described methods (44,45). A detailed content comparison between the current and previous versions of ASD is provided in Table 1. The most exciting enhancement in ASD v3.0 is the number of allosteric modulators. Currently, ASD v3.0 contains 71538 allosteric modulators consisting of 25339 activators, 33604 inhibitors and 13462 regulators, which have increased by >200% since the ASD v2.0 update. The definition of the three classes was provided in our previous publication (44). This increase is primarily a result of the significant expansion of allosteric drug discovery. Among the increased number of allosteric modulators, 10.5% bind to new allosteric targets that have been discovered within the last 2 years, 2.80% were found to have more than one allosteric target and 1.43% were found to regulate allosteric targets with different allosteric effects, dual activators/regulators (0.58%), dual inhibitors/regulators (0.54%), dual activators/inhibitors (0.28%) and multiple activators/inhibitors/regulators (0.03%). Importantly, regardless of the potential off-target binding to orthosteric sites, the growth of modulators interacting with multiple allosteric sites from ∼1.5% in ASD v2.0 to nearly 3% in ASD v3.0 reveals a potentially dangerous situation about the so-called selectivity of allosteric sites.

**Table 1.** Data statistics for allosteric proteins and modulators in updated ASD 3.0^1^

Data category	ASD 3.0	ASD 2.0
**Number of all modulators**	71 538	22 003
Number of activators	25 339	15 144
Number of inhibitors	33 604	6205
Number of regulators	13 462	854
Number of dual activators/regulators	334	50
Number of dual inhibitors/regulators	320	55
Number of dual activators/inhibitors	257	125
Number of multiple activators/inhibitors/regulators	44	30
**Number of all allosteric sites**	1930	907
**Number of all proteins**	1473	1261
Number of kinases	207	187
Number of GPCRs	118	109
Number of ion channels	134	119
Number of peptidases	59	55
Number of phosphatases	30	27
Number of transcription factors	55	46
Number of nuclear receptors	26	24
Number of E-proteins	5	5
Number of other proteins	839	689
**Number of protein–modulator interactions**	75 462	23 120
**Number of all protein pathways**	56	48
**Number of allosteric related diseases**	3350	565

^1^The number of allosteric proteins in ASD 3.0 was counted as the sum of all reported species of each allosteric protein.

In addition to the substantially increasing number of allosteric modulators, we have also increased the associated allosteric features and annotations, including more interactions (∼226% increase), more sites (∼113% increase), more pathways (∼17%) and a greater number of related diseases (∼493% increase) (Table 1). As the identification of allosteric sites increases, proteins with up to four allosteric sites are observed in ASD v3.0. For example, both *Human* α-amylase (46) and glycogen phosphorylase (47) can be regulated by endogenous/exogenous modulators on four distinct allosteric sites. Due to the key roles of glycogen phosphorylase in the process of anti-diabetes, various classes of inhibitors were discovered on each of the four allosteric sites and the combination of inhibitors on different sites demonstrate synergistic effects on the enzyme activity (48). These findings indicate a novel strategy of synergistic regulation on allosteric drug design by targeting more than one allosteric site on the same protein.

## NEW FEATURES AND FUNCTIONALITIES

### Web interface and access

**Figure 1.**
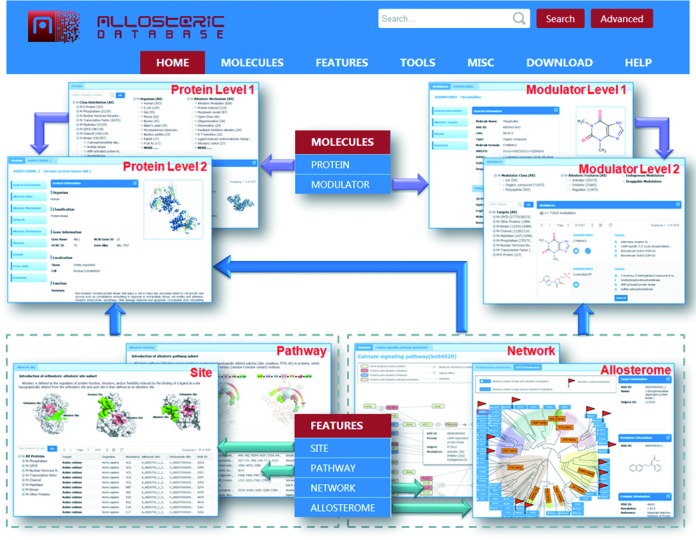
The new web interface and data architecture in ASD 3.0 are depicted.

With collaboration with interested users, we have designed a completely new web interface for ASD v3.0 that is more user-friendly and better integrated (Figure 1). Allosteric data were restructured into three categories with clearly demarcated menus ‘MOLECULES’, ‘FEATURES’ and ‘DOWNLOAD’. The ‘MOLECULES’ menu provides allosteric proteins in ‘PROTEIN’ and allosteric modulators in ‘MODULATOR’, where all molecules are curated by experimental confirmation and annotated with allosteric features. The new flat layout of ‘PROTEIN’ and ‘MODULATOR’ pages allows streamlined access to the data. We believe this should make the data easier to view and browse. Allosteric molecules and related annotations are also downloadable from the ‘DOWNLOAD’ menu for off-line study. In the ‘FEATURES’ menu, there are four auxiliary allosteric datasets that were originally identified by literature and then computationally generated, including conventional ‘SITE’ for binding pockets of allosteric modulators and ‘PATHWAY’ for remote communications from allosteric site to orthosteric site, newly introduced ‘NETWORK’ for allosteric signal propagations in interconnected cellular pathways and ‘ALLOSTEROME’ for the relationship of allostery in a protein family (see below).

In addition to the data, several computational tools were enhanced and added to allow efficient access to the ASD. First, through text-mining design based on the characters of allosteric information, the precision and time of search engine in ASD v3.0 was heavily improved and more accurate search results can be retrieved by performing a quick ‘Search’ or ‘Advanced’ search in the upper right corner of ASD home page. Second, interactive classification trees and filters were added to the browse pages of allosteric data in ‘PROTEIN’, ‘MODULATOR’, ‘SITE’ and ‘NETWORK’, allowing users to precisely restrict the browse by multiple options. Third, an internal crosslink between different datasets in ASD was carefully generated, such as links from nodes in ‘NETWORK’ and ‘ALLOSTEROME’ to entries in ‘PROTEIN’ or ‘MODULATOR’, integrating the knowledge of allosteric data by sequence, structure, evolution and cellular functions. Fourth, all java-based plugins in the previous version of ASD were replaced with javascript codes, making the visualization of the data much simpler and much faster. Fifth, the high-quality data of allosteric sites in ASBench was integrated in ASD for the development of efficient algorithms on the prediction of unknown allosteric sites, which is located in the ‘TOOL’ menu of ASD (49,50). ‘MISC’ is a collection of allosteric knowledge, research and applications, such as ‘GLOSSARY’, ‘DIAGRAM’, ‘LITERATURE’, ‘DRUG’ and ‘EXPERT’, which have been built in the previous version and carefully refined in the current version. Finally, the new web interface has been extensively tested to support Firefox 5+, Chrome 9+, Safari 4+ and Internet Explorer 11 and more FAQs can be found in the ‘HELP’ menu of the ASD homepage.

### Allosteric modulator action

Bioactive modulators can exploit functional pockets in protein targets to exert action on regulation. Unlike orthosteric modulators, for which the key mechanism of small molecule action is trying to compete with the natural ligand in active site, in the case of allosteric modulators, the extent of conformational transitions into functional state can be pivotal in specifying the modulator action in allosteric sites (23). Identification of the origin of the conformational transitions in protein by the modulator is highly dependent on comparing the structure of the allosteric sites before binding (*apo* structure) with that after binding (*holo* structure) (23,51,52), which could not only account for the underlying mechanism of specific allosteric triggering action but may also be used to improve the triggering efficacy in allosteric drug design. Ruth *et al*. proposed an alignment strategy to successfully illustrate a series of allosteric modulator action in the regulation of regulatory subunit of protein kinase A (allosteric agonist: cAMP), Ras (allosteric agonist: GTP) and PDK1 (allosteric agonist: P47, etc.) (23). Likewise, using allosteric *apo*/*holo* structural alignment, we investigated the action of endogenous adenosine triphosphate (ATP) as a modulator in distinct allosteric sites of 13 proteins and found that a majority of the allosteric sites (80%) were induced by the triphosphate in ATP as a trigger, whereas a smaller number of allosteric sites (20%) were triggered by the adenine in ATP (53). These studies showed a fundamentally different mechanistic foundation of allosteric modulator action compared with the orthosteric modulator. To better understand modulator mechanism in allosteric site, a large-scale dataset revealing allosteric modulator action is more desirable than ever and was thus built in the ASD in the current release.

**Figure 2.**
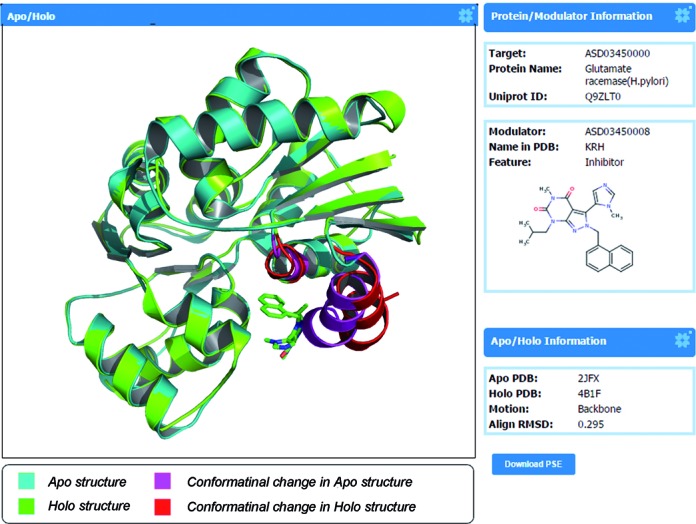
Conformational changes of the allosteric site comparing the protein structure of *apo* and *holo*. Example of Glutamate racemase in *Helicobacter pylori* (ASD03450000_1) with the binding of the allosteric modulator ‘KRH’ (ASD03450008).

In ASD v3.0, 1688 allosteric *apo*/*holo* paired structures for allosteric modulator action in 308 proteins from 107 organisms were constructed using the same protocol described in ATP action (53) (see Supporting Information). All allosteric actions for a protein are listed in the ‘Allosteric Mechanism’ section of the protein page from the ‘PROTEIN’ submenu of ‘MOLECULES’ in the ASD home page. Clicking the modulator button under an action of interest in the list, a new window opens to show details of the *apo*/*holo* structure pair with basic information, including the bound allosteric modulator, the source of the *apo* and *holo* structures and the extent of the conformational transition, *etc*. As shown in Figure 2, the superimposition of allosteric *apo*/*holo* paired structures in ‘Glutamate racemase (*Helicobacter pylori*)’ is displayed. Conformational transitions in allosteric site upon binding of the corresponding allosteric modulator ‘KRH’ are identified in ‘backbone’ and highlighted in color. The alignment and identified conformational transitions can also be downloaded as a PyMOL session (PSE) file in the page for further analysis. Of the 1688 allosteric *apo*/*holo* paired structures, 92.6% and 5.9% allosteric sites showed local motions in backbone and in sidechain, respectively upon the binding of modulators. Remarkably, 20 allosteric proteins can be regulated by the actions of both agonism and antagonism on the same allosteric site, such as glutamate dehydrogenase (bovine) (54,55). The *apo*/*holo* results revealed that the opposite mechanisms into one allosteric site occur by triggering different hotspots of the site, raising great challenges in allosteric drug design.

### Allosteric network

**Figure 3.**
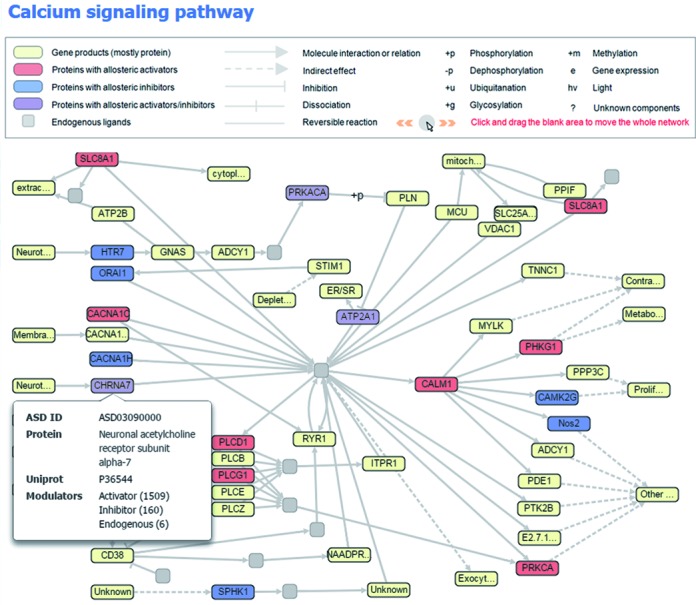
Example of the allosteric network ‘Calcium signaling pathway’.

Because proteins function through a molecular network that is highly interconnected by cellular pathways, a single allosteric action on the given protein in the network can strengthen, weaken or switch the signal propagation toward a specific pathway, triggering various favorable or unfavorable destinies of the cell (56). More generally, cellular functions are determined and dynamically regulated by multiple co-occurring allosteric actions in the network. An illustration of the allosteric map at the network level is highly useful to understand how allostery controls physiological and pathological effects by endogenous/exogenous action and can subsequently reveal critical targets for allosteric related diseases (57,58). To integrate the dynamic regulation of allosteric proteins and reveal their relationships in functional networks, great efforts have been made to build allosteric networks through the Kyoto Encyclopedia of Genes and Genomes (KEGG) reference pathway maps (59,60) (see Supporting Information). In terms of experimental allosteric knowledge, 261 allosteric networks, in which allosteric proteins function as perturbed nodes and allosteric modulators act as perturbants, were built through crosslinking and manual calibration. The networks can be accessed under the ‘NETWORK’ submenu of ‘FEAUTRES’ in the ASD home page. By clicking the selected network, the details of allosteric actions in the network are shown in a separate window. As an example, ‘Calcium signaling pathway’ in Figure 3 demonstrates that the network contains 19 known allosteric proteins, where 13 and 11 can be regulated to produce enhanced and reduced signals to downstream partners for the cellular function. More importantly, endogenous allosteric ligands were found in 13 targets, such as ‘Phosphorylase kinase (activator: cAMP)’ (61), ‘Protein kinase Cα (activator: arachidonic acid)’ (62), ‘5-hydroxytryptamine receptor 7 (inhibitor: *c*-9,10-octadecenoamide)’ (63), ‘Phospholipase Cγ1 (activator: phosphatidic acid)’ (64) and ‘1-phosphatidylinositol 4,5-bisphosphate phosphodiesterase δ1 (activator: glycerophosphoinositol)’ (65), based on published literature, suggesting that normal physiological effect of Ca^2+^ concentration in the cell is achieved by a complicated balance of these endogenous allosteric regulations and that the dysregulation of allostery could disrupt the balance into a pathological state and related disease.

### Allosterome

**Figure 4.**
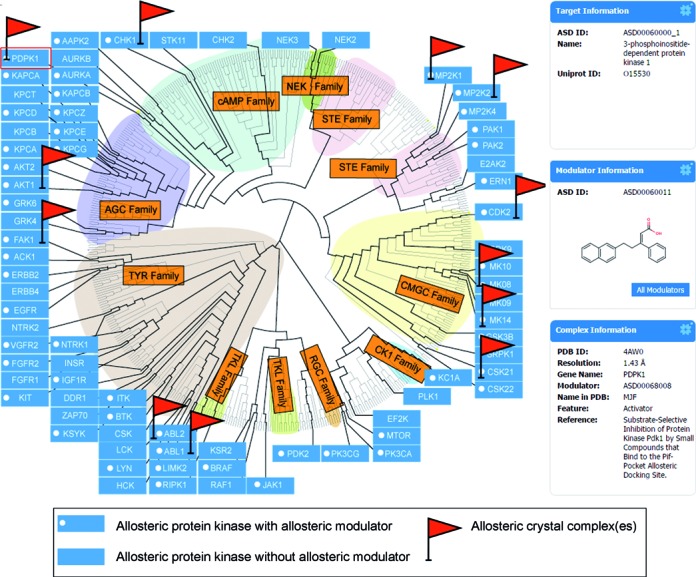
The allosteric distribution in the protein kinase allosterome.

Allosteric sites, which differ from highly conserved orthosteric sites, offer intrinsic opportunities for drug target by providing higher selectivity, fewer side effects and lower toxicity (7,66,67). Analysis of the ASD collection reveals that protein kinases and GPCRs (68) are two of the largest families of allostery that have been successfully exploited in drug discovery. Dissection of the map of the less evolutionary conserved allosteric sites in both families could not only reveal the interrelatedness and specificity among the sites, but be used to guide the rational discovery of allosteric sites in other challenging families, such as nuclear receptors, ion channels and transcription factors, etc. In ASD v3.0, two allosteromic maps in humans, protein kinases and GPCRs, were thoroughly constructed based on multiple sequence alignment of sequences and dendrogram (see Supporting Information) under the menu of ‘ALLOSTEROME’ in the ASD home page. In Figure 4, the allosteric modulators of 51 of 76 human allosteric protein kinases have been discovered (white circle), and the binding allosteric sites of 12 of these modulators have been resolved using X-ray crystallography and nuclear magnetic resonance (red flag). These sites actually cluster into nine locations throughout classical kinase structures in the family. Likewise, all five allosteric sites in human GPCRs show three different locations by structural superimposition. These results indicate that despite the high specificity of allosteric sites, the location of novel sites could still be inferred by the known sites of the same family.

## CONCLUSION AND FUTURE DIRECTIONS

The ASD is a comprehensive and integrated allosteric platform that consolidates manually curated chemical, biological and clinical data about all experimentally confirmed allosteric evidences. Building on previous versions, we have significantly expanded the allosteric molecules and features, particularly modulators, sites, interactions and related diseases, which reflect the recent considerable growth of allosteric studies in both pharmaceuticals and medicine. We also introduced three novel datasets for the investigation of allosteric mechanism including (i) allosteric modulator action; (ii) allosteric network and (iii) protein kinase and GPCR allosteromes in this update. The ASD site was completely redesigned, with greatly improved performance of data access, a cleaner appearance and improved usability. In the future, we aim to continue to curate allosteric data from the primary biomedical literature and to refine the quality of the data according to the research progress of allostery. The ASD will also continue to improve database architecture for easy and intuitive use based on the response from community. In addition to our current knowledge, we will focus on introducing allosteric evolution by interspecies navigation and comparison in order to trace to the beginning of allostery. With the increasing number of clinical mutations discovered in patients using next-generation sequencing, the effects and mechanism of the mutations around the allosteric site could provide new insights in syndromes, physiological abnormalities and diseases, and this is another aspect in which we are planning to curate in the future.
